# Chromosome-level genome assembly of *Nemipterus japonicus* based on PacBio sequencing and Hi-C technology

**DOI:** 10.1038/s41597-025-06536-x

**Published:** 2026-01-06

**Authors:** Feng-Ling Lei, Ren-Xie Wu, Fan-Li Long, Fang-Yuan Han, Zhen-Bang Liang

**Affiliations:** College of Fisheries, Guangdong Ocean University, Zhanjiang, 524088 China

**Keywords:** Ichthyology, Genome-wide association studies

## Abstract

*Nemipterus japonicus* is an economically important demersal fish species inhabiting warm coastal waters of the Indo-West Pacific region, yet its genomic resources remain scarce. Here, we successfully assembled a high-quality chromosome-level reference genome of *N. japonicus* by integrating Illumina, PacBio, and Hi-C sequencing technologies. The genome assembly spans 723.27 Mb, with a contig N50 of 30.83 Mb, and 97.04% of the contigs were anchored onto 22 pseudochromosomes, yielding a scaffold N50 of 30.96 Mb. A total of 23,514 protein-coding genes and 6,532 non-coding RNAs were predicted, with repetitive sequences accounting for 21.84% of the genome. Comparative genomic analysis revealed that *N. japonicus* has 218 expanded and 1,076 contracted gene families, with the divergence times of 56.9 to 85.9 million years ago from three Sparidae species. Positive selection analysis further identified 163 positively selected genes potentially involved in growth and development, reproduction, immune response, and DNA repair of *N. japonicus*. This study provides fundamental data for investigating the genetic evolution of *N. japonicus* and the genomes of other Nemipterid fish.

## Background & Summary

Japanese threadfin bream (*Nemipterus japonicus*) is a representative species of the family Nemipteridae (Perciformes), a commercially important demersal fish widely distributed in the warm coastal waters of the Indo-West Pacific region^1^. In the southeastern coastal waters of China, *N. japonicus* is among the key target species for bottom trawling and longline fisheries, with particularly high abundance in the northern South China Sea (NSCS)^2^. Its annual catch (approximately 50,000 tons) accounts for about 15–20% of the total yield of *Nemipterus* species in the NSCS. *N. japonicus* has a high nutritional value, a delicate flavor, and contains substantial proteins, omega-3 fatty acids, and various vitamins and minerals in its muscle tissue^3^. Ecologically, it occupies a mid-to-high trophic level in marine food webs, feeding on small invertebrates and fishes^4,5^, while also serving as a crucial prey for higher-level carnivorous fish^6^. These attributes underscore its pivotal role in fisheries resources and ecosystems across the Indo-West Pacific region.

Current researches on *N. japonicus* encompass multiple disciplines, including morphological characteristics^1,7^, growth traits^8,9^, feeding ecology^10,11^, reproductive biology^12,13^, population dynamics^14,15^, and phylogenetics^16–19^. Previous studies indicated that *N. japonicus* exhibits typical r-selected life history traits characterized by rapid growth, high fecundity, prolonged spawning periods with extensive spawning ground distribution, and variable feeding behaviors, highlighting its strong ecological plasticity. Molecular phylogenetic analyses revealed two distinct geographic lineages of *N. japonicus*: Indian Ocean and Northwest Pacific populations, with clear phenotypic differentiation in abdominal coloration (yellow vs. silver-white), suggesting potential genetic differentiation associated with cryptic species^20,21^. Along the Indian coast, the eastern population shows pronounced sexual growth dimorphism, a pattern less marked in the western population^8^. The northern population displayed allometric growth patterns, and the southern population followed an isometric growth pattern^22^. Additionally, the primary spawning season for *N. japonicus* in the Bay of Bengal (November to March) significantly differs from other Indo-West Pacific populations (May to October)^21^. The diversity and endemicity observed in reproductive biology, ecological adaptability, and population genetic structure of *N. japonicus* suggest that it may possess unique and complex genomic features. However, only the mitochondrial genome data of this species have been reported so far^23^, with no published information on the whole genome. Thus, in-depth research on its adaptive evolutionary mechanisms, population genetic structure, evolutionary history, and germplasm resource conservation remains severely limited.

This study integrated Illumina Nova-Seq, PacBio SMRT sequencing, and Hi-C chromosome conformation capture technology to conduct deep sequencing and assembly of the *N. japonicus* genome, aiming to obtain high-quality chromosome-level genomic data. First, the RNA-seq data from Illumina and PacBio platforms were used to systematically annotate repetitive sequences, the structure and function of protein-coding genes, and non-coding RNAs (ncRNAs) in the genome by combining homology, *de novo*, and RNA-seq predictions. Second, comparative genomic analyses were performed between *N. japonicus* and 18 other fish species, including gene family clustering, expansion and contraction analysis, phylogenetic tree construction, and divergence time estimation, with synteny analysis using coding and whole-genome sequences of the closely related species, *Sparus aurata*. Finally, eight positive selection groups were established based on the morphological traits of *N. japonicus* for enrichment analysis and gene functional annotation to identify potential trait-associated genes. Thus, this study became the first whole-genome sequencing and assembly of a species in the family Nemipteridae, providing a genomic foundation for evolutionary biology, germplasm resource conservation, and sex-determination mechanism analyses of *N. japonicus*. Moreover, it serves as a reference genome for genetic investigation of other Nemipterid fish.

## Methods

### Sample collection and nucleic acid extraction

**Fig. 1:**
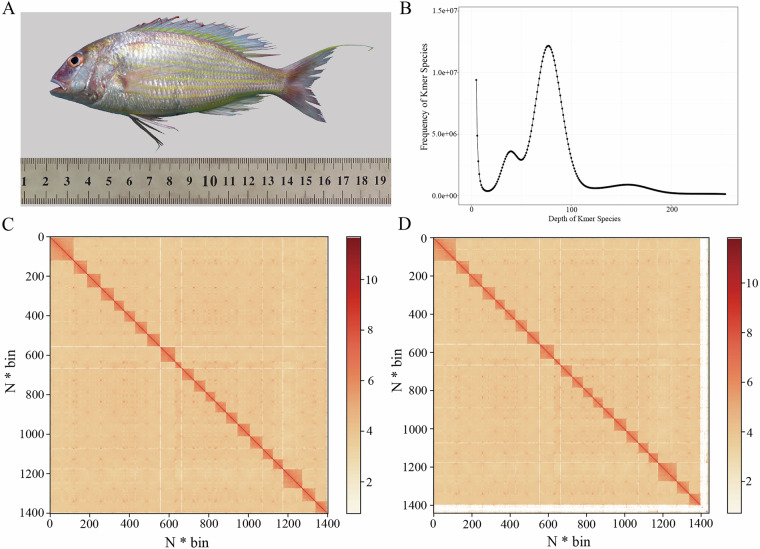
Specimen, genome size estimation, and chromosome-level assembly for *N. japonicus* genome sequencing. (**A**) Specimen used for genome sequencing. (**B**) Genome-size estimation based on 17-mer distribution. (**B**) The 17-mer distribution for the genome-size estimation. (**C**)~(**D**) Chromosome (**C**) and genome-wide (**D**) heat maps at 500 kb resolution of Hi-C assembly of *N. japonicus*. The 500 kb corresponds to 1 bin and the color bar indicates the logarithm of the strength of the contact density.

A live *N. japonicus* (specimen ID: 20210923002) (Fig. 1A) captured in the coastal waters of Maoming City, Guangdong Province, China, in September 2021, was used for genomic sequencing. The specimen measured 14.5 cm in body length and weighed 80.4 g. After anesthesia with MS-222 solution (200 mg/L) (ethyl 3-aminobenzoate methanesulfonate, Sigma-Aldrich, Shanghai, China), the muscle, liver, heart, and brain tissues were quickly excised and separately stored in 1.5 mL sterile tubes. The tissues were flash-frozen in liquid nitrogen and transferred to a −80 °C refrigerator in the laboratory. Genomic DNA was extracted from the muscle tissue using the phenol/chloroform method^24^, while RNA was extracted from the above four tissues using Trizol reagent (Invitrogen, Carlsbad, CA, USA).

### Library construction and genome sequencing

A paired-end sequencing library with a 350 bp insert size was constructed following the standard Illumina experimental protocol^25^ and sequenced on the Illumina NovaSeq-6000 platform (Illumina, San Diego, CA, USA), generating 489,256,470 raw reads, 73.39 Gb of data (Table 1). The raw data were deduplicated and filtered using FASTQ (v0.23.2)^26^, resulting in 428,627,422 clean reads (63.60 Gb) with a 40.8% GC content (Table 1). Concurrently, a 15 kb-insert SMRTbell library was constructed using the SMRTBell Template Prep Kit 1.0 (Pacific Biosciences, Menlo Park, CA, USA) and sequenced on the PacBio Sequel II platform (Pacific Biosciences, Menlo Park, CA, USA) in CCS (Circular Consensus Sequencing) mode. The raw sequencing data were adapter-trimmed and filtered to remove adapter sequences. After multiple sequence alignment and consensus correction using SMRTlink^27^, 2,449,579 high-precision HiFi reads generated 35.63 Gb of data (Table 1). The HiFi reads had an average length of 14,544 bp and an N50 length of 14,749 bp, with a 41.32% GC content.

**Table 1:** Sequencing data statistics of the *N. japonicus* genome assembly and annotation.

Type	Platform	Library (bp)	Raw data (Gb)	Clean data (Gb)	Coverage (×)
Illumina Nova	Illumina NovaSeq-6000	350	73.39	63.60	106.36×
PacBio SMRT	PacBio Sequel II	15k	35.63	—	49.26×
Hi-C	Illumina HiSeq 2500	350	75.80	72.96	108.20×
Illumina RNA-seq	Illumina NovaSeq-6000	350	7.46	7.00	10.31×
ONT RNA-seq	NanopromethION	—	14.03	13.40	19.40×

### Genome size estimation and assembly

A K-mer analysis (K = 17) was performed using Jellyfish (v1.11.1)^28^ on Illumina sequencing-derived clean data, revealing a 17-mer depth of 77 (Fig. 1B). The estimated *N. japonicus* genome size was 700 Mb, and later adjusted to 690 Mb, and the genome heterozygosity rate was 0.56%. Subsequently, HiFi reads were rapidly assembled using Hifiasm (v0.16.1)^29^. The initially assembled genome was de-redundant with Purge Haplotigs (v1.0.4)^30^, yielding 257 contigs with maximum, N90, and N50 lengths of 53,928,558 bp, 23,744,612 bp, and 30,830,163 bp, respectively (Table 2). The *N. japonicus* genome size (based on contig-level assembly) was 723.27 Mb with GC content of 41.46%.

**Table 2:** Sequence statistics of the *N. japonicus* genome assembly.

Level	Contig/Scaffold Length(bp)	Contig/Scaffold Number
N90	23,744,612	21
N80	26,166,573	18
N70	27,417,517	15
N60	29,042,877	13
N50	30,830,163	10
Total length	723,269,530	—
Number (≥ 100 bp)	—	257
Number (≥ 2 kb)	—	257
Max length	53,928,558	—

### Hi-C library preparation, sequencing, and chromosome assembly

First, the sticky ends generated from muscle tissue DNA after cross-linking with paraformaldehyde solution (Sbjbio, Nanjing, China) and restriction enzyme Mbo I digestion were end-repaired using biotin labeling. Subsequently, adjacent DNA fragments were ligated using DNA ligase, followed by protease treatment to reverse cross-linking. The purified DNA was then randomly fragmented into 300–500 bp fragments. Finally, the biotin-labeled DNA fragments were captured using streptavidin magnetic beads, followed by end repair, A-tailing, adapter ligation, PCR amplification, and purification to complete the Hi-C library construction.

The qualified library was sequenced using paired-end sequencing on the Illumina HiSeq 2500 platform (Illumina, San Diego, CA, USA), yielding 505,351,562 raw reads (75.80 Gb). After quality control, 496,771,290 clean reads (72.96 Gb) were obtained (Table 1), with a 41.84% GC content. Further quality control and filtering using HiCUP (v0.8.0)^31^, 186,013,178 generated valid read pairs (accounting for 97.18% of all read pairs), representing 74.89% of total sequencing data. Next, deduplication resulted in 130,388,169 valid pairs (68.12%), accounting for 52.49% of the total sequencing data (Table 3).

**Table 3:** Statistics of the *N. japonicus* HiCUP filtering.

Di-Tag Type	Di-Tag Count	Percent in Paired(%)	Percent in Total Reads(%)
Same Circularised	317,783	0.17	0.13
Same Fragment Dangling Ends	787,768	0.41	0.32
Same Fragment Internal	2,357,537	1.23	0.95
Re-ligation	1,933,771	1.01	0.78
Contiguous Sequence	0	0.00	0.00
Wrong Size	0	0.00	0.00
Invalid Pairs	5,396,859	2.82	2.17
Valid Pairs	186,013,178	97.18	74.89
Valid Pairs (de-duplication)	130,388,169	68.12	52.49

The contigs were partitioned, anchored, ordered, oriented, and merged with 3D-DNA software^32^ to achieve a chromosome-level genome assembly. The interaction map of the assembled genome was constructed using Juicer (v1.6)^33^, then corrected and visualized in JuiceBox (v1.11.08)^34^ to rectify chromosomal translocations and inversions. The resulting interaction map conformed to the principles of chromosomal interactions (Fig. 1C,D), confirming the effectiveness of Hi-C-assisted assembly. Ultimately, 97.04% of the sequences were successfully anchored onto 22 pseudochromosomes, with a total chromosome length of 700.57 Mb (Table 4). The contig N50 and scaffold N50 of the *N. japonicus* genome assembly were 30.83 Mb and 30.96 Mb, respectively (Table 4).

**Table 4:** Statistics of the *N. japonicus* Hi-C-assisted assembly.

Type	Sequence length (bp)	Sequence number	Contig N50 (bp)	Scaffold N50 (bp)
Raw	723,269,530	257	30,830,163	30,830,163
Hi-C	721,909,522	152	30,830,163	30,963,390
Hi-C chr	700,571,217	22	30,830,163	30,963,390
Hi-C nochr	21,338,305	130	304,857	465,076

### RNA library construction and transcriptome sequencing

First, mRNA purified from the mixed RNA samples was reverse transcribed using the TUREscript First Strand cDNA Synthesis Kit (AidLab, Beijing, China) to synthesize double-stranded cDNA libraries. An Illumina paired-end sequencing library with ~350 bp insert size was constructed. Subsequently, the library was sequenced on the Illumina NovaSeq-6000 platform (Illumina, San Diego, CA, USA), yielding 7.46 Gb of raw data and 7.00 Gb of clean data after filtering (Table 1). Furthermore, a Nanopore full-length transcriptome library was prepared using the PCR Barcoding (SQK-PBK004) and PCR-cDNA Sequencing Kits (SQK-PCS109, Oxford Nanopore Technologies, Oxford, UK). Sequencing was performed on the PromethION sequencer (Oxford Nanopore Technologies, Oxford, UK), generating 14.03 Gb of raw data and 13.40 Gb of clean data after filtering (Table 1).

### Genome annotation

**Fig. 2:**
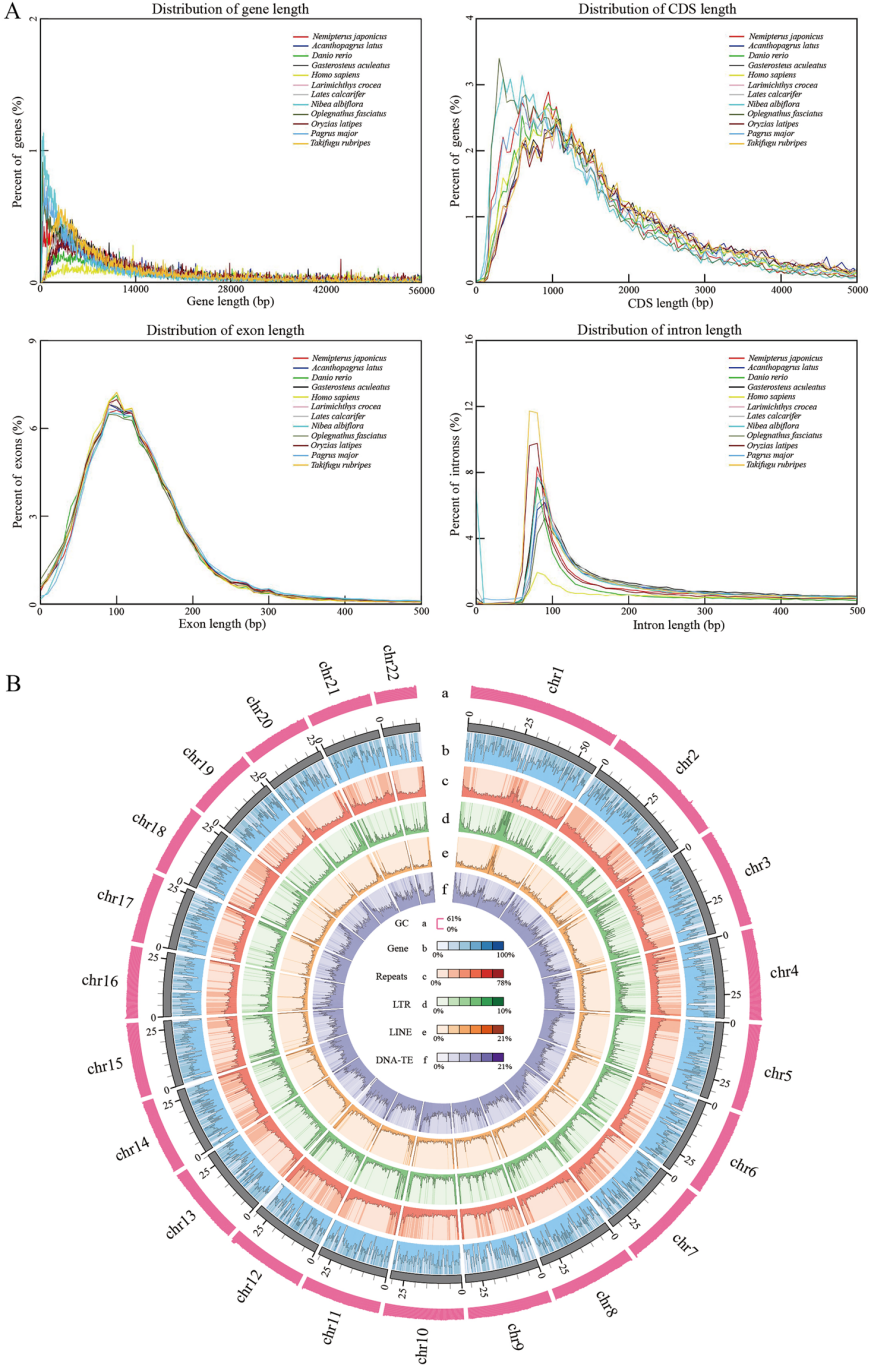
The *N. japonicus* gene elements length distribution and genomic characteristics circle figure. (**A**) The comparisons of different gene elements in the *N. japonicus* genome with other fish species. (**B**) Circle figure of the genomic characteristics of *N. japonicus*, including (a) the GC content of the genome, (b) the distribution of genes, (c) the distribution of repeats, (d) the distribution of long tandem repeats, (e) the distribution of long interspersed nuclear elements, and (f) the distribution of DNA transposable elements.

Gene annotation in *N. japonicus* was performed by integrating homology-based prediction, *de novo* prediction, and RNA-seq/EST-based prediction methods. First, the whole-genome sequences of 11 reference species, including *Homo sapiens*, *Danio rerio*, *Oryzias latipes*, *Lates calcarifer*, *Larimichthys crocea*, *Nibea albiflora*, *Pagrus major*, *Acanthopagrus latus*, *Oplegnathus fasciatus*, *Gasterosteus aculeatus*, and *Takifugu rubripes*, were downloaded from the NCBI database. The genome sequence of *N. japonicus* was aligned with the protein-coding sequences of these 11 species using tblastn (E-value ≤ 1 × 10-5)^35^ and Genewise (v2.4.1)^36^ to predict the gene structure of *N. japonicus*. Second, Augustus (v3.3.2)^37^ was utilized to calculate the codon usage frequencies, exon-intron distributions, and other factors. Third, RNA-seq data were assembled to obtain transcripts using Tophat (v2.1.1) and Cufflinks (v2.2.1)^38^. Finally, the three gene sets were integrated into a non-redundant, more complete gene set using Maker2 (v2.31.10)^39^. After incorporating the CEGMA results and employing the HiCESAP workflow, 23,514 protein-coding genes were predicted. The average lengths of the predicted genes, coding sequences (CDS), exons, and introns were 16,089 bp, 1,732 bp, 274.90 bp, and 1,451 bp, respectively. Their distribution in the *N. japonicus* genome is highly consistent with that of the 11 reference species (Table 5, Fig. 2A), reflecting their evolutionary conservation. *N. japonicus* has 23,514 protein-coding genes, all of which were aligned to the NR (Non-Redundant Protein), SwissProt (Swiss Protein Institute), KEGG (Kyoto Encyclopedia of Genes and Genomes), TrEMBL (Translation of European Molecular Biology Laboratory), and GO (Gene Ontology) databases for gene functional annotation. Moreover, the PFam (Protein Family) and InterPro (Integrated Resource of Protein) databases were employed to predict conserved functional domains and protein family information. Ultimately, 22,858 genes (97.21%) were successfully annotated, while 656 genes (2.79%) remained unannotated (Table 6).

**Table 5:** Structure and parameters of the predicted genes in the *N. japonicus* genome.

Methods	Gene set	Protein coding gene number	Average gene length (bp)	Average CDS length (bp)	Average exon per gene	Average exon length (bp)	Average intron length (bp)
*De novo*	AUGUSTUS	33,606	9885.41	1255.69	6.95	180.64	1450.06
Homolog	*Nibea albiflora*	29,648	10,506	1,151	6.20	185.53	1,798
	*Danio rerio*	34,108	13,533	1,391	7.65	181.67	1,825
	*Homo sapiens*	26,288	13,518	1,288	7.97	161.49	1,754
	*Pagrus major*	41,578	11,955	1,327	7.06	188.04	1,755
	*Oplegnathus fasciatus*	39,067	11,230	1,266	7.22	175.22	1,601
	*Gasterosteus aculeatus*	33,579	12,773	1,453	8.07	179.95	1,601
	*Oryzias latipes*	33,257	12,953	1,475	7.97	185.00	1,646
	*Lates calcarifer*	35,248	13,702	1,453	7.99	181.90	1,753
	*Takifugu rubripes*	30,883	12,913	1,481	8.33	177.82	1,560
	*Larimichthys crocea*	36,198	13,053	1,445	7.84	184.37	1,697
	*Acanthopagrus latus*	37,639	14,102	1,456	7.81	186.40	1,856
BUSCO		3,673	12,098	1,852	12.03	153.92	928.81
MAKER		24,806	15,033	1,697	9.81	263.82	1,412
HiCESAP		23,514	16,089	1,732	10.16	274.90	1,451

**Table 6:** Functional annotation of the genes in the *N. japonicus* genome.

Type	Number	Percent (%)
Total	23514	—
Annotated	22858	97.21
InterPro	20722	88.13
GO	15729	66.89
KEGG_ALL	22465	95.54
KEGG_KO	14409	61.28
Swissprot	19931	84.76
TrEMBL	22630	96.24
TF	3574	15.2
Pfam	19944	84.82
NR	22800	96.96
KOG	18472	78.56
Unannotated	656	2.79

Repetitive sequence annotation was performed by combining homologous sequence alignment and *de novo* prediction. First, RepeatMasker (v4.1.2)^40^ and RepeatProteinMask (v4.1.2)^41^ were run against the RepBase database^42^ to identify sequences homologous to known nucleic acid and amino acid repetitive sequences, respectively. Second, a *de novo* repetitive sequence library was constructed using LTR-Finder (v1.0.7)^43^, RepeatModeler (v2)^44^, and RepeatScout (v1.0.5)^44^, followed by repetitive sequence prediction with RepeatMasker (v4.1.2)^40^. The integration of both methods predicted 157,659,348 bp of repetitive sequences in *N. japonicus*, accounting for 21.84% of its genome. Further, tandem repetitive sequences predicted by TRF (v4.10.0)^45^ generated 42,505,501 bp (5.89% of the genome). Overall, the proportions of repetitive DNAs, LINEs, SINEs, and LTRs in the *N. japonicus* genome were 10.55%, 3.54%, 0.33%, and 2.33%, respectively (Table 7). Additionally, the distribution of protein-coding genes, repetitive sequences, LTR, LINE, and DNA-TE across the 22 pseudochromosomes of *N. japonicus* is illustrated in Fig. 2B.

**Table 7:** Statistics of repetitive sequences in the *N. japonicus* genome.

Type	RepBase TEs		TE Protein		De novo		Combined TEs	
	Length (bp)	% in genome	Length (bp)	% in genome	Length (bp)	% in genome	Length (bp)	% in genome
DNA	42,114,538	5.83	4,203,692	0.58	45,617,87	6.32	76,192,342	10.55
LINE	15,411,205	2.13	8,890,320	1.23	14,480,114	2.01	25,527,614	3.54
SINE	1,563,033	0.22	0	0.00	1,233,439	0.17	2,373,014	0.33
LTR	11,685,076	1.62	3,357,687	0.47	6,674,779	0.92	16,826,206	2.33
Satellite	4,728,176	0.65	0	0.00	8,395,252	1.16	10,138,320	1.40
Simple repeat	0	0.00	0	0.00	90,356	0.01	90,356	0.01
Other	7,119	0.00	831	0.00	0	0.00	7,950	0.00
Unknown	796,836	0.11	14,589	0.00	49,243,903	6.82	49,687,176	6.88
Total	68,173,879	9.44	16,449,475	2.28	112,695,424	15.61	157,659,348	21.84

The non-coding RNAs (ncRNAs) were also annotated. tRNAs were predicted using tRNAscan-SE (v2.0)^46^. The rRNA sequences of closely related species were used as references to identify rRNAs of *N. japonicus* through BLAST alignment. miRNA and snRNA sequences were predicted using Infernal (v1.1.4)^47^ integrated with Rfam (v1.1.4)^48^. Thus, 6,532 non-coding RNAs were detected in the *N. japonicus* genome, comprising 894 miRNAs (77,062 bp; 0.010675%), 2,240 tRNAs (168,686 bp; 0.023367%), 2,215 rRNAs (269,715 bp; 0.037361%), and 1,183 snRNAs (179,014 bp; 0.024797%) (Table 8).

**Table 8:** Annotation statistics of the non-coding RNAs in the *N. japonicus* genome.

Type		Copy number	Average length(bp)	Total length(bp)	% in genome
miRNA		894	86	77,062	0.010675
tRNA		2,240	75	168,686	0.023367
rRNA	rRNA	2,215	122	269,715	0.037361
	18S	0	0	0	0.000000
	28S	0	0	0	0.000000
	5.8S	190	154	29,258	0.004053
	5S	2,025	119	240,457	0.033308
snRNA	snRNA	1,183	151	179,014	0.024797
	CD-box	167	119	19,869	0.002752
	HACA-box	76	149	11,300	0.001565
	splicing	932	157	145,939	0.020216
	scaRNA	8	238	1,906	0.000264

### Gene family clustering, expansion, and contraction analyses

**Fig. 3:**
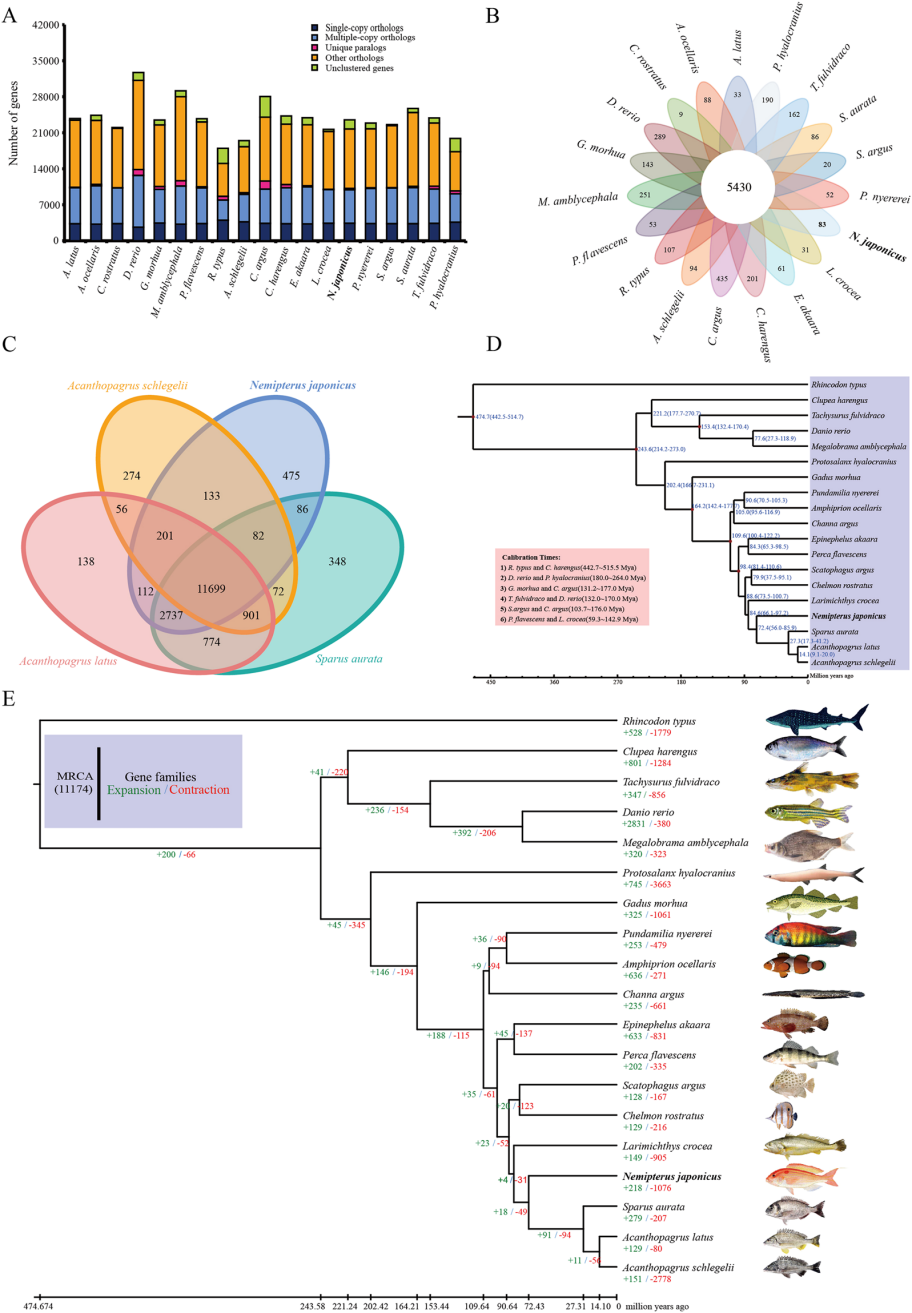
The *N. japonicus* phylogenetic position and genome evolution. (**A**) Statistics of the orthologous gene families in 19 fish species. (**B**) Cluster petal map of gene families of 19 fish species. (**C**) Venn diagram of the number of homologous gene families in the four species. (**D**) Phylogenetic analysis and divergence time tree of *N. japonicus* and 18 other fish species. (**E**) Phylogenetic tree and gene family contractions and expansions for the 19 fish species. Green (+) and red (−) numbers represent the number of expanded and contracted gene families, respectively.

The genomes of 18 fish species were downloaded from NCBI for gene family clustering and phylogenetic analyses, including *Rhincodon typus*, *D. rerio*, *Megalobrama amblycephala*, *Gadus morhua*, *Clupea harengus*, *Protosalanx hyalocranius*, *Tachysurus fulvidraco*, *Perca flavescens*, *Epinephelus akaara*, *A. latus*, *Acanthopagrus schlegelii*, *S. aurata*, *L. crocea*, *Scatophagus argus*, *Amphiprion ocellaris*, *Chelmon rostratus*, *Channa argus*, and *Pundamilia nyererei*. First, protein-encoding genes with < 30 amino acids or containing internal stop codons were removed from the genomes of these 18 species and *N. japonicus*, retaining only the longest transcript in the coding region. Then, all-vs-all blastp (v2.11.0+)^49^ was used to establish similarity relationships between protein sequences of all species (E-value = 1e-5). OrthoMCL (v2.0.9)^50^ (inflation coefficient = 1.5) was subsequently employed to cluster the 23,514 protein-coding genes of *N. japonicus* into 15,525 gene families. Among these, all 19 species shared 5,430 gene families, while 83 were unique to *N. japonicus*. The 19 species had 1,666 single-copy genes (Fig. 3A,B). Additionally, comparative clustering analysis between *N. japonicus* and three Sparidae species (*S. aurata*, *A. latus*, and *A. schlegelii*) revealed 11,699 shared gene families and 475 unique gene families for *N. japonicus* (Fig. 3C).

**Fig. 4:**
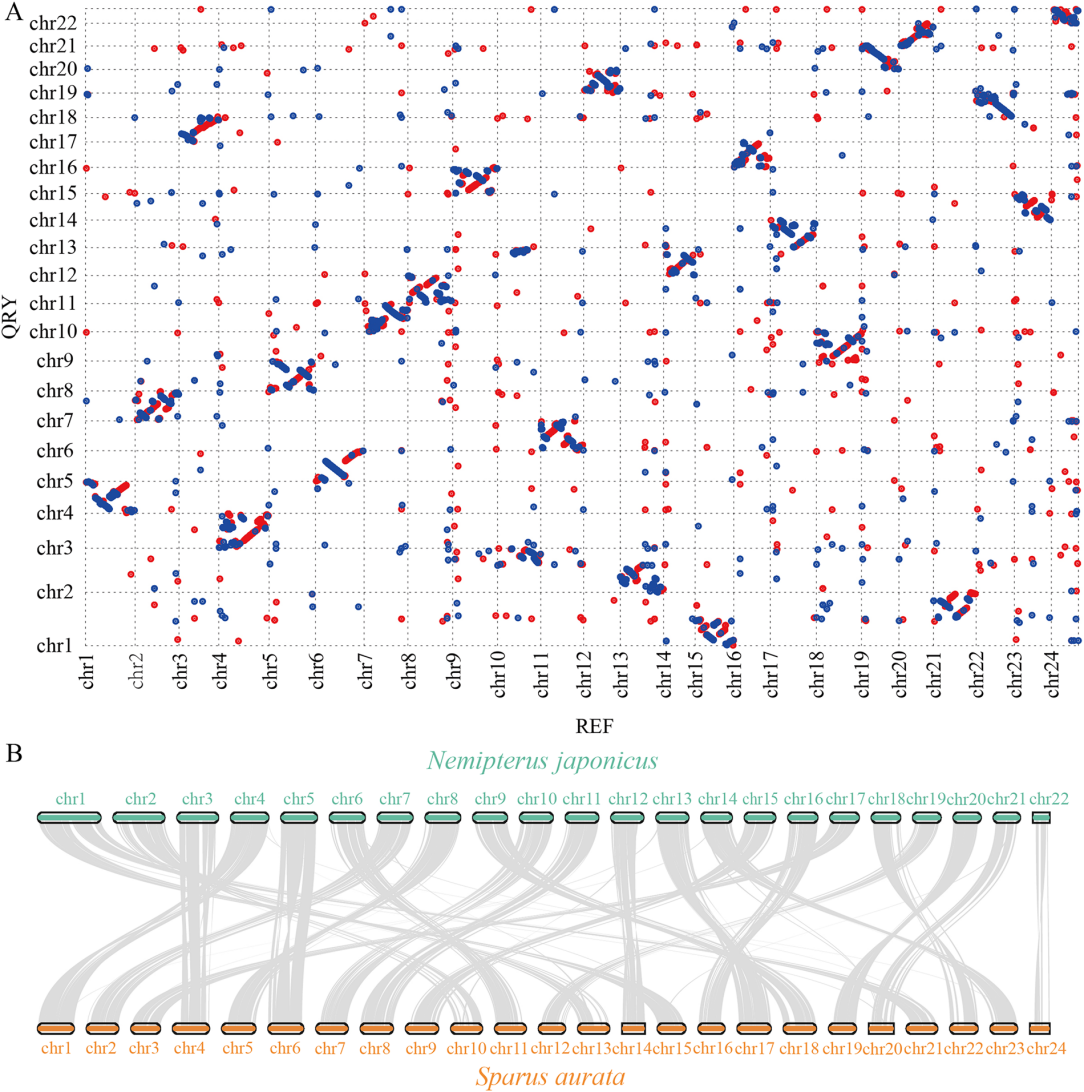
Collinearity analyses of *N. japonicus* and *S. aurata* based on genome sequence (**A**) and coding sequence (**B**).

The *N. japonicus* gene family expansion and contraction analyses using CAFÉ5 (v5.0.0)^51^ revealed 218 expanded and 1,076 contracted gene families (Fig. 3E). KEGG and GO enrichment analyses were performed on the 302 genes from 43 significantly expanded gene families and 190 from 42 significantly contracted gene families. The genes from the expanded gene families primarily enriched pathways such as neurodegeneration-multiple diseases, Huntington disease, calcium signaling pathway, and amyotrophic lateral sclerosis. The genes from the contracted gene families mainly enriched the parathyroid hormone synthesis, secretion, and action, NOD-like receptor signaling pathway, cGMP-PKG signaling pathway, and adrenergic signaling in cardiomyocytes. Synteny analyses between *N. japonicus* and *S. aurata* were performed using Mummer (v4.0.0rc1)^52^ (based on whole-genome sequence) and JCVI (v1.1.22)^53^ (based on coding sequence); the results demonstrated strong chromosomal collinearity between the two species (Fig. 4A,B).

### Phylogenetic analysis

Protein sequences from each single-copy gene family across the 19 species were aligned using MAFFT (v7.487)^54^, and the maximum-likelihood phylogeny was inferred using RAxML (v8.2.12)^55^. Next, six calibration points were selected from the Timetree database^56^, including the divergence time between *R. typus* and *C. harengus* (442.7–515.5 Mya), *D. rerio* and *P. hyalocranius* (180.0–264.0 Mya), *G. morhua* and *C. argus* (131.2–177.0 Mya), *T. fulvidraco* and *D. rerio* (132.0–170.0 Mya), *S. argus* and *C. argus* (103.7–176.0 Mya), and *P. flavescens* and *L. crocea* (59.3–142.9 Mya). The divergence times among the 19 species were then estimated using McMcTree (v4.9) in PAML^57^. The ML tree clustered *N. japonicus* with three Sparidae species (*S. aurata*, *A. latus*, and *A. schlegelii*), with their divergence time estimated at 56.9–85.9 million years ago (Mya) (Fig. 3D), earlier than the divergence time among the three Sparidae species (17.3–41.2 Mya).

### Positive selection analysis

This study established eight positive selection groups for genome-wide comparative analysis to identify candidate genes potentially linked to phenotypic traits in *N. japonicus*. Group 1 (*N. japonicus*, *A. ocellaris*, and *P. nyererei*) vs. (*M. amblycephala*, *A. schlegelii*, *P. hyalocranius*, and *C. harengus*) was designed to identify genes associated with the bright-red body coloration of the species. Group 2 (*N. japonicus*, *A. latus*, *P. flavescens*, *L. crocea*, and *T. fulvidraco*) vs. (*M. amblycephala*, *A. schlegelii*, *P. hyalocranius*, and *C. harengus*) and Group 3 (*N. japonicus*) vs. (*A. latus*, *P. flavescens*, *L. crocea*, *T. fulvidraco*, and *Sinocyclocheilus grahami*) were further screened for genes related to the yellow coloration and its position on the body surface of *N. japonicus*. Group 4 (*N. japonicus*, *D. rerio*, *P. nyererei*, *A. ocellaris*, and *P. flavescens*) vs. (*E. akaara*, *S. grahami*, *S. argus*, and *C. argus*) focused on genes linked to striped or spotted body patterns. Group 5 (*N. japonicus*, *D. rerio*, and *T. fulvidraco*) vs. (*A. ocellaris*, *P. flavescens*, *C. rostratus*, *A. schlegelii*, and *P. nyererei*) targeted genes responsible for longitudinal or transverse body bands. Group 6 (*N. japonicus*, *A. latus*, *S. aurata*, and *A. schlegelii*) vs. (*C. argus*, *G. morhua*, *L. crocea*, *E. akaara*, and *S. argus*) examined caudal fin shape-related genes. Group 7 (*N. japonicus*, *A. latus*, and *A. schlegelii*) vs. (*C. harengus*, *G. morhua*, and *D. rerio*) investigated genes related to the presence or absence of fin spines. Group 8 (*N. japonicus*) vs. (*A. latus*, *A. schlegelii*, and *S. aurata*) detected positively selected genes between *N. japonicus* and its closely related species. Positive selection acting on protein-coding sequences was detected using CodeML (v4.9) in PAML^58^ based on the branch-site model^59^, revealing 163 positively selected genes, which were subjected to KEGG and GO enrichment analyses and visualization. Pathways and terms meeting the criteria of P < 0.05 and FDR < 0.05 were filtered to explore the functions of these genes. Consequently, the main KEGG-enriched pathways included cytokine-cytokine receptor interaction, base excision repair, Fanconi anemia pathway, homologous recombination, mismatch repair, and Th1 and Th2 cell differentiation.

A combined functional analysis of positive selection genes with the biological traits of the species, identified several genes related to growth and development, interleukins, and DNA repair that have undergone significant evolution in the *N. japonicus* genome (Table S1). Specifically, the *LEPR*, *GHR*, *PRLR*, *PDGFD*, *BMP9*, *BMP14*, and *FOS* genes appear to be significantly involved in growth and skeletal development, lipid metabolism, and reproductive processes. The interleukin-related genes are dominated by the IL gene family, including *IL1B*, *IL1R1*, *IL1R2*, *IL1RAP*, *IL6ST*, *IL10RA*, *IL12B*, *IL12RB2*, *IL13RA2*, *IL17RB*, *IL17RC*, *IL17C*, and *IL17F*. DNA damage repair pathways include genes involved in interstrand cross-link repair (*FANCB*, *FANCD2*, *FANCL*, *FANCI*, and *UBE2T*), mismatch repair (*EXO1*, *RFC2*, *MLH3*, *PMS2*, and *SSBP1*), double-strand break and homologous recombination repair (*BRCA1*, *MRE11*, *NBN*, *RAD51D*, *RAD52*, *RAD54B*, *BRIP1*, *EME1*, *MUS81*, *SLX1A*, and *SLX4*), and translesion synthesis (*POLD3*, *POLE*, *POLH*, *POLK*, *REV1*, *PARP1*, *PARP2*, *PARP4*, and *XRCC1*).

### Ethics declarations

The experimental animal protocols in the present study were reviewed and approved by the Animal Experimental Ethics Committee of Guangdong Ocean University, China (approval number: 202108001). Experiment procedures were performed in accordance with the Provisions and Regulations for the National Experimental Animal Management Regulations (China, July 2013) and the Experimental Animal Policies and Regulations of Guangdong Province (China, October 2010).

## Data Records

The genome assembly data of *N. japonicus* has been deposited in public repositories^60,61^. The DNA and RNA sequencing data are archived in the Sequence Read Archive (SRA) under BioProject number PRJNA1256535 and BioSample number SAMN48153383, with SRA accession number SRR33394854^62^, SRR33398644^63^, SRR33393141^64^, SRR33394373^65^, SRR33406548^66^ and SRR33419198^67^. The genome annotation and comparative genomic analyses files have been deposited in figshare^68,69^.

## Technical Validation

### Quality assessment of genomic DNA and RNA

The concentration, purity, and integrity of the extracted DNA were assessed using Qubit 3.0 fluorometer (Invitrogen, Carlsbad, CA, USA), Nanodrop 2000 microvolume spectrophotometer (Thermo Fisher Scientific, Waltham, MA, USA), and Agilent 4200 Bioanalyzer (Agilent Technologies, Palo Alto, CA, USA), respectively. The integrity and concentration of extracted RNA were detected using Agilent RNA ScreenTape Assay (Agilent Technologies, Palo Alto, CA, USA) and Agilent 4200 TapeStation (Agilent Technologies, Palo Alto, CA, USA). Equal amounts of RNA from four qualified tissues were pooled and rechecked for integrity, concentration, and purity. Subsequently, the high-quality DNA and pooled RNA samples that passed quality control were used for library preparation and sequencing.

### Evaluation of genome assembly integrity

The genome sequences were fragmented and aligned to the NCBI nucleotide database using blastn (v2.11.0+)^70^ to confirm that the assembly result belonged to the target species. Illumina and PacBio sequencing data were then aligned to the assembled genome using the BWA (v0.7.12)^71^ and Minimap2 (v2.22)^72^ software, respectively. Alignment rates reached 99.35% with 99.97% coverage for Illumina clean reads, and 99.70% with 99.80% coverage for PacBio HiFi reads. A joint plot of GC content versus coverage depth distribution analysis showed a stable GC content of around 41.46% without significant GC bias, indicating no exogenous contamination in the genome assembly. Variants in the genome were identified using Samtools (v1.9)^73^, Picard (v1.124) (https://broadinstitute.github.io/picard/), and GATK (v4.2.0.0)^74^. The observed homozygous SNP (0.000%) and InDel (0.001%) rates were extremely low, indicating high assembly accuracy. Finally, the integrity of genome assembly based on the BUSCO (Benchmarking Universal Single-copy Orthologs method^75^ was evaluated with Augustus (v3.3.2)^37^ using 3,640 orthologous single-copy genes as references. The results revealed 3,558 (97.75%) complete BUSCO genes in the *N. japonicus* genome, including 3,505 (96.29%) complete single-copy and 53 (1.46%) complete duplicated genes (Table 9), demonstrating high gene coverage.

**Table 9:** The completeness and accuracy of the *N. japonicus* genome assembly and annotation.

Type	Genome		Annotation	
	Proteins	Percentage (%)	Proteins	Percentage (%)
Complete BUSCOs (C)	3,558	97.75	3,507	96.3
Complete and single-copy BUSCOs (S)	3,505	96.29	3,459	95.0
Complete and duplicated BUSCOs (D)	53	1.46	48	1.3
Fragmented BUSCOs (F)	6	0.16	36	1.0
Missing BUSCOs (M)	76	2.09	97	2.7
Total BUSCO groups searched	3,640	100	3,640	100

## Supplementary information

Supplementary Table S1.

## Data Availability

The genome assembly data of *N. japonicus* has been deposited to the National Center for Biotechnology Information (NCBI) GenBank at JBNJRZ000000000 (https://identifiers.org/ncbi/insdc.gca:GCA_053574625.1)^60^ and figshare (10.6084/m9.figshare.30630179)^61^. The genomic Illumina sequencing data were available in the Sequence Read Archive at SRR33394854 (https://identifiers.org/ncbi/insdc.sra:SRR33394854)^62^. The genomic PacBio sequencing data were available in the SRA at NCBI SRR33398644 (https://identifiers.org/ncbi/insdc.sra:SRR33398644)^63^. The RNA-seq data were available in the SRA at NCBI SRR33393141 (https://identifiers.org/ncbi/insdc.sra:SRR33393141)^64^ and SRR33394373 (https://identifiers.org/ncbi/insdc.sra:SRR33394373)^65^. The Hi-C sequencing data were available in the SRA at NCBI SRR33406548 (https://identifiers.org/ncbi/insdc.sra:SRR33406548)^66^ and SRR33419198 (https://identifiers.org/ncbi/insdc.sra:SRR33419198)^67^. The genome annotation files were available in figshare (10.6084/m9.figshare.29061083)^68^. The protein sequences used in the comparative genomic analyses and the output files including gene family clustering, and expansion and contraction analyses, and positive selection analysis were also available in figshare (10.6084/m9.figshare.29063051)^69^.

## Code availablility

No specific code was used in this study. All data analyses were performed using standard bioinformatics tools. The commands and parameter settings for each tool were executed according to their official manuals and established pipelines. Detailed descriptions have been provided in the Methods section.
